# Light scattering in stacked mesophyll cells results in similarity characteristic of solar spectral reflectance and transmittance of natural leaves

**DOI:** 10.1038/s41598-023-31718-1

**Published:** 2023-03-22

**Authors:** Kai Xu, Hong Ye

**Affiliations:** Department of Thermal Science and Energy Engineering, University of Science and Technology of China, Hefei, 230027 People’s Republic of China

**Keywords:** Plant sciences, Plant physiology, Biological physics

## Abstract

Solar spectral reflectance and transmittance of natural leaves exhibit dramatic similarity. To elucidate the formation mechanism and physiological significance, a radiative transfer model was constructed, and the effects of stacked mesophyll cells, chlorophyll content and leaf thickness on the visible light absorptance of the natural leaves were analyzed. Results indicated that light scattering caused by the stacked mesophyll cells is responsible for the similarity. The optical path of visible light in the natural leaves is increased with the scattering process, resulting in that the visible light transmittance is significantly reduced meanwhile the visible light reflectance is at a low level, thus the visible light absorptance tends to a maximum and the absorption of photosynthetically active radiation (PAR) by the natural leaves is significantly enhanced. Interestingly, as two key leaf functional traits affecting the absorption process of PAR, chlorophyll content and leaf thickness of the natural leaves in a certain environment show a convergent behavior, resulting in the high visible light absorptance of the natural leaves, which demonstrates the PAR utilizing strategies of the natural leaves. This work provides a new perspective for revealing the evolutionary processes and ecological strategies of natural leaves, and can be adopted to guide the improvement directions of crop photosynthesis.

Leaf is the organ of natural plants that absorbs sunlight for photosynthesis^1–3^. When a collimated light is incident onto the adaxial surface of a leaf, a part of the light is diffusely reflected by the interface with rough structure^4,5^, and the rest penetrates the leaf. A structure of stacked mesophyll cells with air spaces is exist in natural leaves, and the optical parameters of the cytosol, cell wall and air that constitute the structure are significantly different^6^. Therefore, the light entering the leaf is refracted at the interfaces of the cell walls between cytosol and air, resulting in the scattering process of light in the stacked mesophyll cells^7^. In addition to the scattering process, chlorophyll, carotenoid, water and dry matter, as the main components of natural leaves, absorb the incident sunlight^8,9^. It is worth noting that the solar spectrum includes the visible light, which is called photosynthetically active radiation (PAR) in the field of plant physiology^10–12^. Chlorophyll absorbs PAR for photosynthesis and converts light energy into chemical energy^13,14^, while carotenoids are auxiliary pigments involved in the harvesting process of light energy^15,16^. When the light arrives at the abaxial surface of leaves, a part of the light is transmitted out from the interface between the epidermis and air. According to the law of conservation of energy, the sum of reflected, absorbed and transmitted energy is the total energy of the incident radiation. Figure 1a shows the measured results of solar spectral reflectance and transmittance of a *Cinnamomum* leaf as an example^17^. As can be seen, the solar spectral reflectance presents green peak, red edge, near-infrared plateau and water absorption valleys. The green peak at approximately 550 nm is caused by the absorption characteristics of chlorophyll. The chlorophyll of natural leaves is distributed in the mesophyll cells, and mainly includes chlorophyll a and chlorophyll b. Chlorophyll a mainly absorbs red and orange light, while chlorophyll b mainly absorbs blue and violet light. Both of them exhibit weak absorption of green light, thus reflectance is high in the green light waveband, and the reflection peak exists^18,19^. It is worth noting that the reflection characteristics of natural leaves in the visible light waveband are closely related to chlorophyll content, thus the reflectance of vegetation can be obtained by remote sensing detection instruments and the chlorophyll content of leaves can be inversed to monitor the growth state of vegetation^20,21^. The red edge locates in the range from 678 to 765 nm. In this range, the absorption of incident radiation by chlorophyll is gradually weakened, while the scattering process in the stacked mesophyll cells is gradually enhanced, resulting in that the reflectance of natural leaves is significantly increased^22^. The near-infrared plateau locates in the range from 765 to 1300 nm. The formation of this feature is also related to the stacked mesophyll cells in the leaf. The collimated incident light is transformed to diffuse light in the leaf through the scattering process, while the components of the leaves all exhibit weak absorption characteristics in this waveband, thus the region of high reflectance is formed^23^. The water absorption valleys are located near 1450 and 1940 nm, which are related to the absorption characteristics of water within the leaf^24^. Moreover, it can be seen from Fig. 1a that natural leaves exhibit low transmittance characteristic in the visible light waveband. In order to ensure that the natural leaves inside the canopy of vegetation absorb enough PAR for photosynthesis, certain leaf distribution structures are developed, so that sunlight can enter the canopy through the gaps between the outermost leaves of the canopy, and the leaves inside the canopy can receive PAR from the sunflecks to promote the photosynthesis of the entire canopy^25,26^.

**Figure 1:**
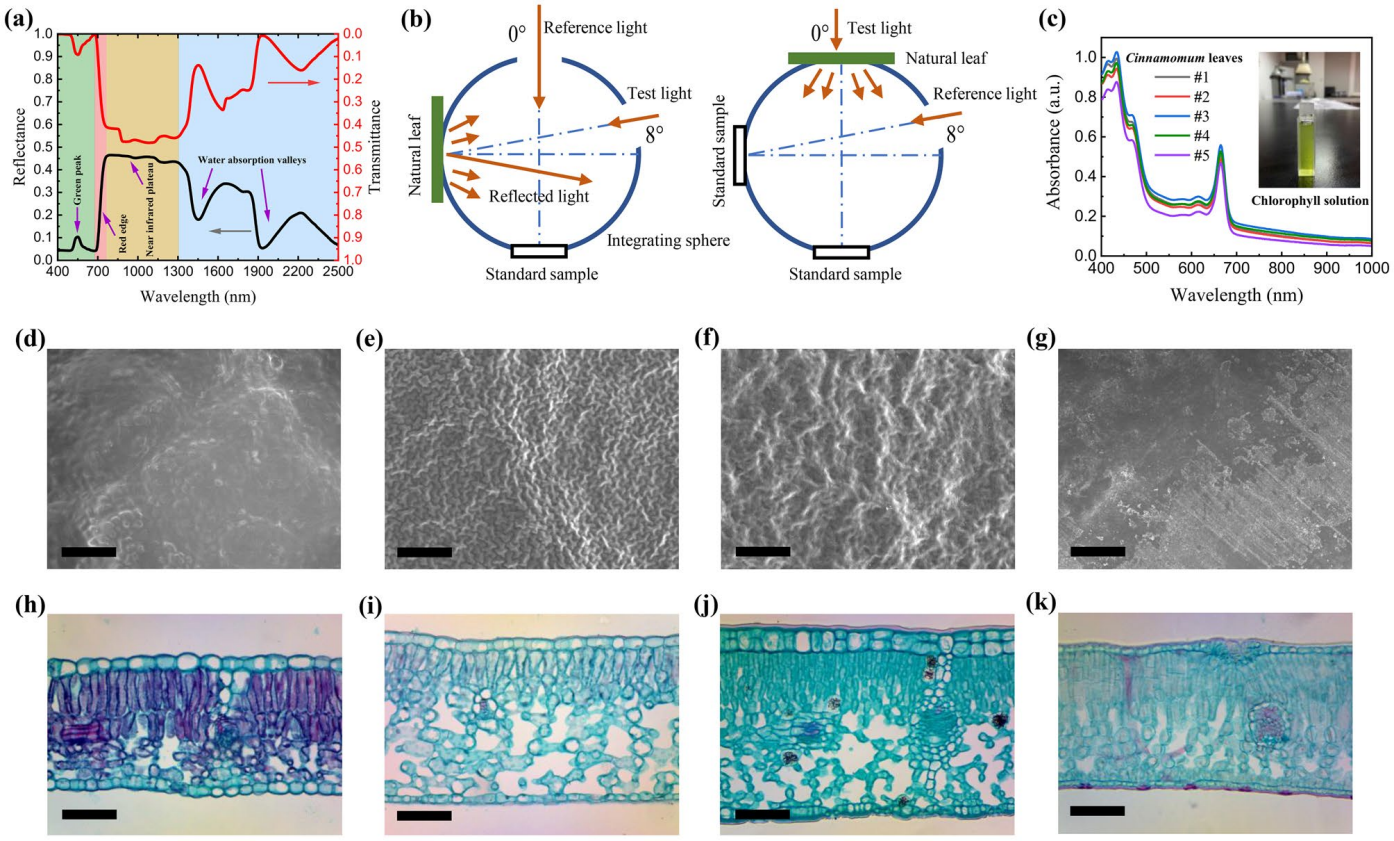
(**a**) Solar spectral reflectance and transmittance of *Cinnamomum* leaves. (**b**) Measurement principles of directional-hemispherical reflectance and directional-hemispherical transmittance. (**c**) Measured results of chlorophyll solution absorbance of *Cinnamomum* leaves. Surface microstructure of the natural leaves, *Cinnamomum* (**d**), *Gardenia* (**e**), *Pittosporum* (**f**), *Osmanthus* (**g**), the scales are all 100 μm. Cross-section microstructure of the natural leaves, *Cinnamomum* (**h**), *Gardenia* (**i**), *Pittosporum* (**j**), *Osmanthus* (**k**), the scales are all 100 μm.

It is worth noting that despite the variety of natural leaves, the solar spectral reflectance and transmittance generally exhibit remarkably similarity^27,28^. Based on the anatomical characteristics of leaves and the law of refraction, Knipling et al. speculated that the similarity is caused by the light scattering in the stacked mesophyll cells in the leaves^22^. In order to clarify the physiological significance of the scattering process, Merzlyak et al. compared the transmission and reflection characteristics of chlorophyll solution and natural leaves^29^. The results show that compared with the chlorophyll solution without scattering process, the light scattering in the stacked mesophyll cells increases the optical path of light in the natural leaves, thereby increasing the visible light absorptance. Photosynthesis is one of the most important physiological processes for plants, and the key procedure in this process is the absorption of visible light by chlorophyll. Visible light absorptance is a key parameter to describe the absorption process. However, the quantitative relationships between visible light absorptance and the two key physiological traits, i.e., stacked mesophyll cells and chlorophyll content, are still unknown. It is worth noting that the rough structure of the leaf surface makes the incident parallel light penetrating the leaf become diffuse light, which promotes the absorption process of visible light in the leaf^30,31^. Moreover, the visible light absorption exhibits distribution characteristics in the thickness direction of leaves^32^. Therefore, when analyzing the formation mechanism and physiological significance of the similarity, it is necessary to consider the diffuse reflection caused by the rough structure of the adaxial leaf surface, and quantitatively analyze the effects of stacked mesophyll cells, chlorophyll content and leaf thickness on the visible light absorptance of natural leaves.

To carry out the analysis mentioned above, a radiative transfer model needs to be constructed to quantitatively describe the reflection, absorption and transmission processes of natural leaves in the solar spectral waveband. Based on leaf spectroscopy, the relationship between the radiation characteristics of leaves and leaf structure and biochemical characteristics can be constructed, and the latter include the content, distribution and absorption characteristics of the components in natural leaves^33,34^. The PROSPECT model is a classic model in the field of leaf spectroscopy to describe the radiation processes of natural leaves in the solar spectral waveband, and is widely utilized in the field of vegetation remote sensing detection^35–39^. This model is based on the physical mechanisms of the interaction of solar radiation with the structure and components of the leaves, the principles of which are the radiation transfer laws followed by the absorption and scattering processes of radiation in the leaves. Noda et al. used the PROSPECT model to reveal the relationship between leaf spectral characteristics and biochemical characteristics of the deciduous plants in the cold zone during seasonal changes, laying a foundation for analyzing remote sensing detection data of plant canopy^40^. Tejada et al. investigated the effects of *Xylella fastidiosa* infection on the spectral characteristics and biochemical characteristics of the crop leaves, and used the PROSPECT model to construct the relationship the two factors, which is of great significance in the field of disease prevention of crops^41,42^. In this work, the PROSPECT model was used to elucidate the formation mechanism and physiological significance of the similarity of solar spectral reflectance and transmittance, and it was found that the light scattering caused by the stacked mesophyll cells of natural leaves makes the visible light absorptance tends to a maximum, promoting the PAR absorption in the natural environments. It is worth noting that Wright et al. found that in order to grow and develop in a certain environment, plants usually trade off resources to meet their own needs to the greatest extent, and based on their findings, a theory named leaf economic spectrum was proposed^43^. The leaf economic spectrum is a combination of a series of leaf functional traits, which are interrelated and change synergistically, and represents the resources utilizing strategies of plants in the form of data. In natural environments, plants may regulate stacked mesophyll cells, leaf thickness, chlorophyll content and visible light absorptance, which are significantly related to the PAR absorption process, and there may be trade-offs among these functional traits. Therefore, the PAR utilizing strategies of natural leaves were also analyzed based on the relationship between these leaf functional traits.

## Experiments and models

### Experiments

#### Sample collection

The collection site of leaf samples locates in Hefei, Anhui Province (31°52′ N, 117°17′ E) in the middle and lower reaches of the Yangtze River, with an average altitude of approximately 20 to 40 m, and belongs to a subtropical monsoon climate zone. The annual average temperature and precipitation are approximately 22 °C and 995.3 mm, respectively, and the midday average solar irradiance density on a sunny day in summer is approximately 800 W/m^2^. The samples including 9 kinds of angiosperms and 1 kind of gymnosperms were selected as typically distributed plants, among which the gymnosperm *Ginkgo Linn.* is known as the living fossil in the plant kingdom. Among the 10 species, *Jasminum nudiflorum* Lindl., *Sebiferum sebifera* (L.) Roxb., *Syringa* Linn. and *Ginkgo biloba* Linn. are deciduous plants, while *Cinnamomum camphora* (L.) J. Presl, *Osmanthus fragrans* (Thunb.) Lour., *Magnolia cylindrica* E.H.Wilson, *Gardenia jasminoides* Ellis, *Hedera nepalensis var. sinensis* (Tobler) Rehder and *Pittosporum* Banks are evergreen plants. The names of the plants were abbreviated to show clear contents in the subsequent figures. The names of these 10 kinds of plants was shortened to *Cinnamomum*, *Osmanthu*, *Magnolia*, *Gardenia*, *Hedera*, *Pittosporum*, *Jasminum*, *Sebiferum*, *Syringa* and *Ginkgo*, respectively. It is worth noting that the chlorophyll content of evergreen plants also changes with seasons and generally reaches a maximum in summer^44^. The young leaves of these 10 kinds of plants were marked in the spring of 2020, and picked in the summer when the leaf samples were mature. The developmental age of the leaf samples was approximately 5 months. Five leaves of each kind of plant were collected from separate individuals for testing, and the total number of testing samples is 50. These leaf samples were considered as representative of the leaves of the plant community in Hefei summer. To avoid the impact of meteorological changes on the structure and components content of the leaves, sample collection was conducted in consecutive days with similar meteorological conditions, and the collection period was from July 12 to July 19. The collected leaves were placed in sealed sample bags and immediately carried to the laboratory for spectrum, composition and structure characterizations avoiding the change of component contents and morphological characteristics. The use of plants or plant parts in the present study complies with international, national and/or institutional guidelines, and we have permissions to collect plant materials from public botanical garden in University of Science and Technology of China. The voucher specimens were stored in University of Science and Technology of China herbarium, with a policy of giving bona fide researchers access to deposited specimens, and that they are scrupulously conserved. The voucher specimens were identified by Mrs. Xiaoyan Liu.

#### Characterizations

The directional-hemispherical reflectance and directional-hemispherical transmittance of the adaxial surface of the samples were measured with an integrating sphere attached to a spectrophotometer DUV-3700. Before the measurement, baseline calibration with the standard sample plate made of BaSO_4_ is required^45^. Figure 1b shows schematic diagrams of measuring the reflectance and transmittance of a natural leaf using an integrating sphere, respectively. The original result $R_{{\text{m}}}$ obtained from the reflectance measurement is the ratio of the reflectance of the natural leaf to the reflectance of the BaSO_4_ plate, and the reflectance of the natural leaves can be calculated according to the reflectance of the BaSO_4_ plate:

1 $$R_{{{\text{leaf}}}} { = }R_{{\text{m}}} \times R_{{{\text{BaSO}}_{4} }}$$

where $R_{{{\text{BaSO}}_{4} }}$ is the reflectance of the BaSO_4_ standard sample^46^. When measuring the transmittance, the test light is collimated along the direction perpendicular to the surface of the natural leaves, and the incident angle is $0^\circ$. It is worth noting that when measuring the transmittance, the standard sample is air, and the transmittance of air in the solar spectrum waveband is approximately 1, thus the original result $T_{{\text{m}}}$ is the transmittance of the natural leaves:

2 $$T_{{{\text{leaf}}}} = T_{{\text{m}}}$$

Considering that the pigments and water contents of the collected leaves would decrease significantly after being placed for a long time^47^, pigments, moisture, and dry matter contents were immediately characterized after the measurments of reflectance and transmittance, and the thickness of the samples were measured with a vernier caliper. In order to measure the pigment contents, the pigments in the leaves were extracted with organic solvent^48^. The sample with an area of $1.5{\text{ cm}} \times 1.5{\text{ cm}}$ was cut into pieces and put in a mortar. Then, a little amount of CaCO_3_, quartz sand and approximately 3 mL of absolute ethanol were added to the mortar, and the mixture was grinded until the leaf tissue turned white. The acidic substances in the cytosol can be neutralized with CaCO_3_ to prevent the pigments from being destroyed^49^, while the function of quartz sand is to destroy the cell structure and make grinding more fully. After fully grinding, the grinding liquid in the mortar was transferred in a beaker, and the volume of the liquid was adjusted to 30 mL with absolute ethanol, resulting in that the absorbance of the liquid at 470, 648.6 and 664.2 nm is between 0.2 and 0.8^50^. In this range, the absorbance value measured by the spectrophotometer is more accurate. After the grinding liquid was statically conserved under shading at room temperature for 24 h, the upper layer of the grinding liquid was extracted and its absorbance was measured with the spectrophotometer. Figure 1c shows the absorbance of the pigment solutions of the five *Cinnamomum* leaf samples as an example. The absorbance of all the sample leaves can be obtained in Supplementary Information 1. The concentration of the pigments in the solution can be calculated from the absorbance^51^:

3 $$c_{{\text{a}}} = 13.36A_{664.2} - 5.19A_{648.6}$$

4 $$c_{{\text{b}}} = 27.43A_{648.6} - 8.12A_{664.2}$$

5 $$c_{{{\text{car}}}} = \frac{{1000A_{470} - 2.13c_{{\text{a}}} - 97.64c_{{\text{b}}} }}{209}$$

where $c_{{\text{a}}}$, $c_{{\text{b}}}$ and $c_{{{\text{car}}}}$ are the concentrations of chlorophyll a, chlorophyll b and carotenoids, respectively, in the unit of mg/L, and *A*_470_, *A*_648.6_ and *A*_664.2_ are the absorbances of the solution at 470, 648.6 and 664.2 nm, respectively. The sum of chlorophyll a and chlorophyll b concentrations is the chlorophyll concentration, and the contents of each kind of pigments in a unit area of leaves can be further calculated using the pigment concentrations. To characterize the water and dry matter contents, the leaf samples with area of $3{\text{ cm}} \times 1{\text{ cm}}$ (Leaf area, *LA*) were weighed, and the masses were recorded as the fresh leaf weight $(W_{{\text{F}}} )$. After weighing, the leaves were placed in an oven at 80 °C for 48 h for fully drying. After drying, the leaves were weighed again to obtain the dry weight $(W_{{\text{D}}} )$. According to the above measured results, the water content and dry matter content per unit area of leaves can be calculated respectively:

6 $$C_{{\text{w}}} \left( {{\text{g/cm}}^{{2}} } \right) = \frac{{W_{{\text{F}}} - W_{{\text{D}}} }}{LA}$$

7 $$C_{{\text{m}}} \left( {{\text{g/cm}}^{{2}} } \right) = \frac{{W_{{\text{D}}} }}{LA}$$

In order to analyze the relationship between radiation transfer processes and the structure of natural leaves, testing samples of surface and cross-section of the collected leaves were prepared and characterized with scanning electron microscope and optical microscopy, respectively. Figures 1d–g show the surface microstructures of the natural leaves by taking *Cinnamomum*, *Gardenia*, *Pittosporum* and *Osmanthus* as examples. It can be seen from Figs. 1d–f that the leaf surfaces exhibit rough structures, thus the radiation incident on the surface undergoes diffuse reflection. Interestingly, it can be seen from Fig. 1g that the surface of *Osmanthus* leaf is relatively flat, because the cuticle of *Osmanthus* leaf is thick^52^. Figures 1h–k shows the cross-sections of the above 4 kinds of leaves. It can be seen that the interior of the natural leaves presents a structure of stacked mesophyll cells, and the geometric scales of the mesophyll cells and intercellular spaces are both in the order of microns. In addition, the thickness of the mesophyll cell wall of natural leaves is generally approximately $10^{ - 1} \, \mu {\text{m}}$^53–55^. Notably, the optical parameters of the cytosol, cell wall, and air that make up the stacked mesophyll cells are significantly different. Woolley showed that the refractive indices of the cytosol and cell wall of fresh soybean leaves are approximately 1.33 and 1.42, respectively, while the refractive index of air in the intercellular spaces is 1^56^. Therefore, when the incident radiation enters the natural leaves, it is refracted at the interfaces of the different components, and then the scattering process occurs within the stacked mesophyll cells. Interestingly, biomineral particles can be observed from the cross-section of the *Pittosporum* leaf in Fig. 1j. The incident radiation can also be scattered by these particles, increasing the optical path and facilitating the absorption process of sunlight in the leaves^57–59^. In the leaf radiation models constructed in Sect. “Leaf radiation models”, the light scattering processes caused by the surface rough structure and stacked mesophyll cells are mainly considered.

### Leaf radiation models

**Figure 2:**
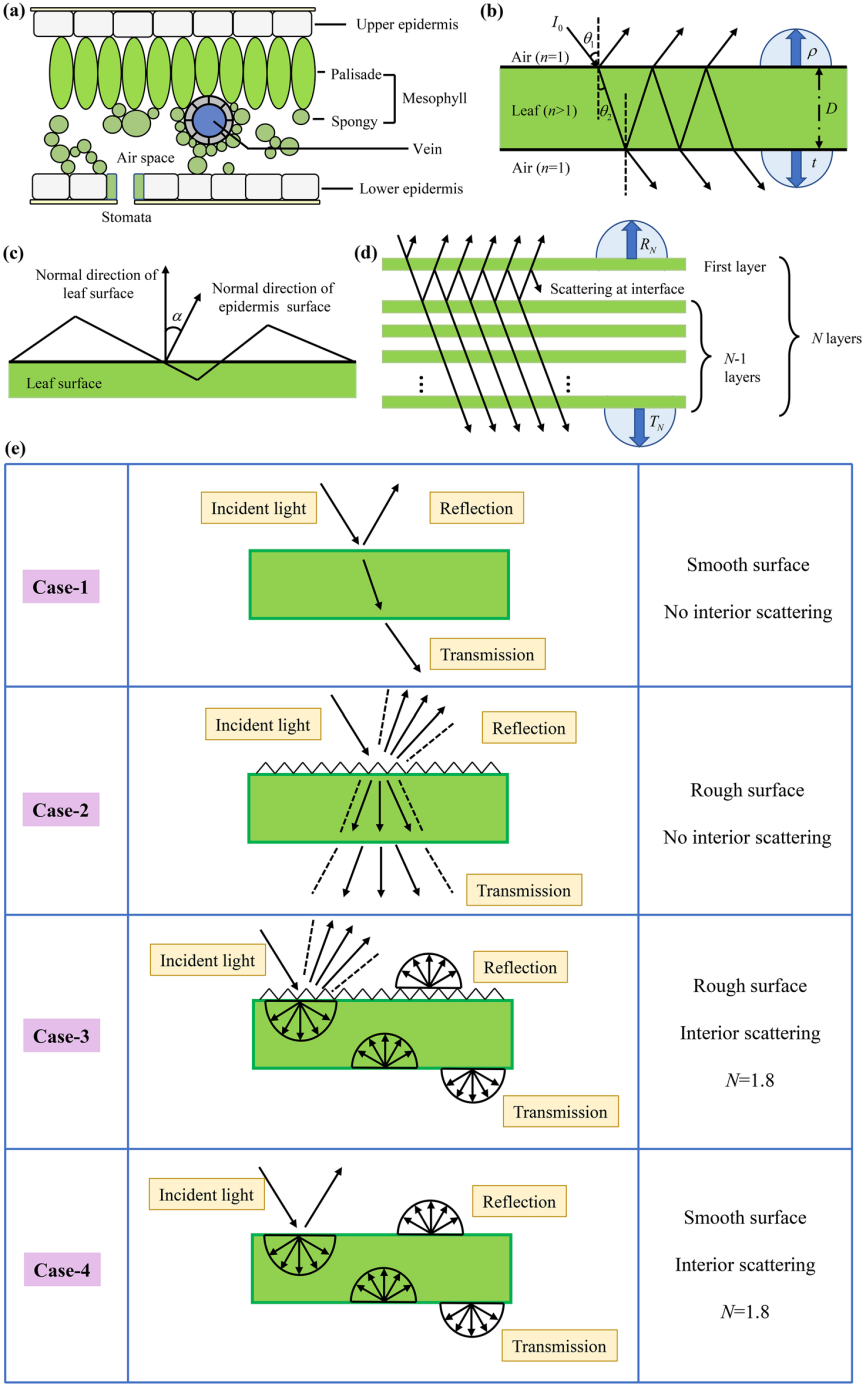
Radiation transfer models of natural leaves, (**a**) schematic diagram of stacked mesophyll cells, (**b**) single-layer plate model, (**c**) schematic diagram of surface rough structure, (**d**) multi-layers plate model (PROSPECT model). (**e**) Cases with differen leaf structures. In Case-3 and Case-4, structural parameter *N* is 1.8.

Figure 2a is a schematic diagram of the cross-section of a natural leaf. In order to elucidate the formation mechanism and physiological significance of the similarity of reflectance and transmittance of natural leaves, it is necessary to construct a radiation model to analyze the relationship between the radiation transfer processes and leaf components and structures. The main idea of PROSPECT model is that the stacked mesophyll cells in the natural leaves is equivalent to a structure of multi-layer plates. The absorption process in the natural leaves can be imitated with the absorption process in the plates, meanwhile the scattering process in the stacked mesophyll cells can be imitated with the interface reflection process between the plates. It is worth noting that the PROSPECT model is developed on the basis of a single-layer plate model, thus this model was introduced firstly.

#### Single-layer plate model

In the single-layer plate model, the leaf is simplified to a dense homogeneous translucent plate with a uniform roughness surface. Figure 2b is a schematic diagram of the interaction between light and a single-layer plate. Air and natural leaves are defined as medium 1 and medium 2, respectively. When the collimated light passes through the medium 1 and is incident on the top surface of the medium 2, a part of the light is reflected by the interface, and the rest penetrates the interior of the medium 2. After undergoing absorption process, the radiation reaches the bottom surface of the medium 2, and the interface reflection and transmission occur again. The reflectance and transmittance of the single-layer plate can be calculated using ray tracing method. The details of the calculation are in Supplementary Information 2. Considering that the upper surfaces of the natural leaves are rough, as shown in Fig. 1d − f, small inclined surfaces are constructed on the upper surface of the single-layer plate model to imitate the interface diffuse reflection, as shown in Fig. 2c. In this case, the incident angle of light is not a fixed value, but is within a certain range^60^, and the specific range is introduced in Sect. “Model parameters”.

#### PROSPECT model

Figure 2a is a schematic diagram of the cross-section of a natural leaf. As can be seen, upper epidermis, palisade tissue, spongy tissue and lower epidermis are the main parts of the natural leaves, of which the spongy tissue presents a loose and porous structure^61,62^. The incident radiation is scattered in the stacked mesophyll cells composed of palisade tissue and sponge tissue, thus it is necessary to improve the single-layer plate model and build a leaf radiation model taking into account the scattering process. The stacked mesophyll cells are simplified as a stack composed of *N* -layer dense and homogeneous flat plates separated by $N - 1$ layers air, as shown in Fig. 2d. The absorption processes in the plates are used to imitate the absorption processes of the main components in the natural leaves, while the interface reflection processes between the plates are used to imitate the scattering process in the stacked mesophyll cells. The interface diffused reflection occurring at the top surface of the first layer can be analyzed with the method introduced in Sect. “Single-layer plate model”. It is noteworthy that the cell walls with rough surfaces also exhibit a diffuse scattering characteristic^63,64^, meanwhile the mesophyll cells show a stacked structure, thus the collimated light entering the leaf transfers to diffused light, and the range of $\theta_{2}$ is adjusted from $0^\circ$ to $90^\circ$ to manifest the scattering effect in the natural leaves. The details of calculating reflectance and transmittance of natural leaves with PROSPECT model were provided in Supplementary Information 2.

#### Model parameters

The PROSPECT model is introduced in Sect. “PROSPECT model”, and the input parameters of the model include *n*, *α*, *N*, $K_{i}$ and $C_{i}$, where *n*, *α* and $K_{i}$ can be obtained according to the literatures, and $C_{i}$ of the components has been obtained through measurements introduced in Sect. “Characterizations”. The undetermined parameter is only *N*. The absorption coefficients $K_{i}$ of each components in the natural leaves and the refractive index *n* of fresh leaves obtained by Feret et al. were used as the input parameters of the PROSPECT model^65^. It is worth noting that the surface of the natural leaves exhibit rough structures, and the parallel incident light transforms into diffuse light after being refracted by the rough surface^66^. Although various kinds of plants exist, the maximum of incident angle $\alpha$ can be set as 40° for most plants^65^. In the field of plant spectroscopy, the layer number *N* is defined as the structure parameter of natural leaves, which can be used to measure the scattering degree of the incident light by the stacked mesophyll cells. Larger structure parameter represents stronger scattering in the leaves. Genetic algorithm toolbox in Matlab was adopted to invert the structure parameters based on the measured results of reflectance and transmittance of the leaf samples^67^. It is worth noting that the total absorption coefficient per unit plate is required when performing the inversion. The components of natural leaves include chlorophyll, carotenoids, dry matter and water, and the total absorption coefficient per unit plate is

8 $$K{ = (}K_{{{\text{ab}}}} \times C_{{{\text{ab}}}} { + }K_{{{\text{car}}}} \times C_{{{\text{car}}}} { + }K_{{\text{m}}} \times C_{{\text{m}}} { + }K_{{\text{w}}} \times C_{{\text{w}}} {)/}N$$

where *K*_ab_ (cm^2^/*μ*g), *K*_car_ (cm^2^/*μ*g), *K*_m_ (cm^2^/g) and *K*_w_ (cm^2^/g) are the absorption coefficients of chlorophyll, carotenoids, dry matter and water, respectively, *C*_ab_ (*μ*g/cm^2^), *C*_car_ (*μ*g/cm^2^), *C*_m_ (g/cm^2^) and *C*_w_ (g/cm^2^) are the corresponding contents of the above components, respectively.

#### Cases design with different leaf structures

After constructing the leaf radiation model and obtaining the model parameters, four cases with different leaf structures were designed to investigate the influences of leaf structural characteristics on the solar spectral reflectance and transmittance, as shown in Fig. 2e. In Case-1, there is a leaf with a smooth surface and no scattering inside. When the parallel light is incident on the adaxial surface, interface reflection and transmission occur at the interface between the air and the leaf epidermis. Because there is no surface rough structure, the light entering the leaf is still parallel, which is partly absorbed by the components in the leaf, and the rest undergoes interface reflection and transmission again on the abaxial surface. The surface rough structure is introduced in Case-2. When parallel light is incident on the adaxial surface, the light transmitting into the leaf is scattered by the surface rough structure into light rays within a certain angle range. Using the single-layer plate model introduced in Sect. “Single-layer plate model”, the reflectance and transmittance corresponding to Case-1 and Case-2 can be calculated. Both surface rough structure and interior scattering structure were introduced in Case-3, which is closest to the real structure of the natural leaves. The structure parameter *N* is greater than 1, and the light entering the natural leaves is scattered by the stacked mesophyll cells into diffuse light. The reflectance and transmittance corresponding to Case-3 can be calculated using the multi-layer plates model introduced in Sect. “PROSPECT model”. Case-4 includes a smooth surface and scattering characteristics inside, which corresponds to the *Osmanthus* leaves. The reflectance and transmittance corresponding to Case-4 can also be calculated using the multi-layer plates model, and the maximum incident angle is set as $0^\circ$. After calculating the reflectance and transmittance corresponding to the four radiation models, the normalized similarity coefficient (SC) according to the Euclidean distance was defined to describe the similarity of the reflectance and transmittance:

9 $$SC{ = }1 - \sqrt {\frac{{\sum\limits_{i = 1}^{m} {(R_{i} - T_{i} )^{2} } }}{m}}$$

where *m* is the number of the discrete wavelengths in the solar spectral waveband when using the spectrophotometer, $R_{i}$ and $T_{i}$ are the measured reflectance and transmittance, respectively. It can be seen that the value range of the similarity coefficient *SC* is in the range from 0 to 1. When *SC* is closer to 0, the similarity is lower. When *SC* is closer to 1, the similarity is higher. It is worth noting that when studying the influence of structure characteristics on the solar spectrum characteristics of the natural leaves, the relationship between solar spectrum characteristics and components of the natural leaves can be investigated by adjusting the component contents in the radiation models, and the work of Li et al. indicates that the average values of component contents in different kinds of leaves can be used to represent the average level of the physiological characteristics of plant communities in a certain environment^68^. Therefore, this method was utilized to describe the leaf functional traits of the various plant samples distributed in Hefei in the following analysis.

### Ethics statement

The use of plants or plant parts in the present study complies with international, national and/or institutional guidelines, and we have permissions to collect plant materials from public botanical garden in University of Science and Technology of China. The voucher specimens are stored in University of Science and Technology of China herbarium, with a policy of giving bona fide researchers access to deposited specimens, and that they are scrupulously conserved. The voucher specimens were identified by Mrs. Xiaoyan Liu. The information of the herbarium deposition was stored in University of Science and Technology of China herbarium, which is belong to Herbarium, Institute of Botany, Academia Sinica (accession number: PE). Digitized information of the herbarium deposition can be accessed through www.cvh.ac.cn.

### Research involving plants

Experimental research and field studies on the plants mentioned in this article complied with relevant institutional guidelines.

## Results and discussion

### Spectral characteristics, structure and component contents

**Figure 3:**
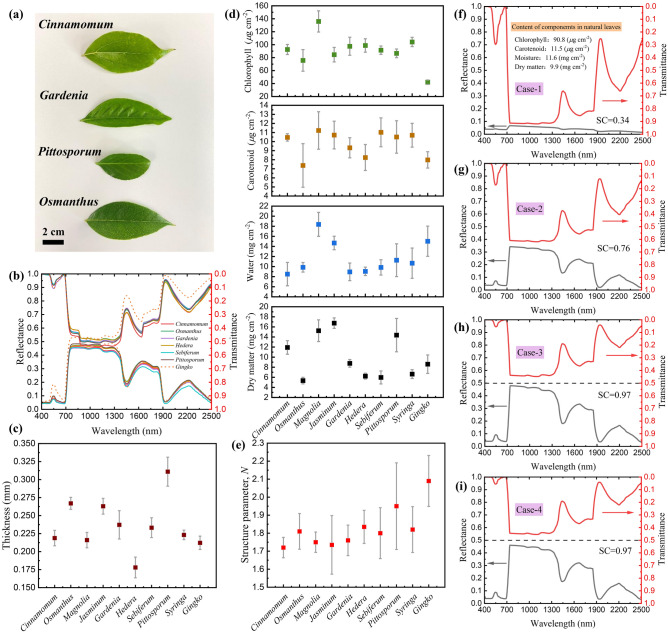
(**a**) Photos of the sample leaves. (**b**) Reflectance and transmittance of the natural leaves. (**c**) Leaf thickness of the natural leaves. (**d**) Contents of the components in the natural leaves, including chlorophyll, carotenoids, water and dry matter. (**e**) Structure parameter of the natural leaves. Calculated reflectance and transmittance of leaf with different structures, Case-1 (**f**), Case-2 (**g**), Case-3 (**h**) and Case-4 (**i**). The similarity coefficient (SC) of reflectance and transmittance is provided.

Figure 3a shows the photos of the natural leaves. It can be seen that the sample leaves are lamellar, and the shapes of the various kinds of leaves are significant different. Figure 3b shows the measured results of the solar spectrum reflectance and transmittance by taking part of the sample leaves as examples. It can be seen from the figure that although there are various kinds of natural leaves, the reflectance and transmittance show significant similar characteristics. This is due to the similar surface and interior structures of the natural leaves, as shown in Fig. 1. According to Eq. (9), it can be known that the similarity coefficients of reflectance and transmittance of the natural leaves are in the range from 0.92 to 0.97. It is worth noting that the reflectance of *Ginkgo* leaves in the visible light waveband is significantly higher, resulting in that the corresponding visible light absorptance is lower. According to the analysis in Sect. “Convergent behavior of chlorophyll content”, it can be known that the characteristic of low visible light absorption is caused by the low chlorophyll content in *Ginkgo* leaves. Figure 3c shows the test results of leaf thickness. As can be seen, the thicknesses of most natural leaves are in the range from 0.2 to 0.3 mm. *Hedera* leaves are thinner, with an average thickness of approximately 0.178 mm, while *Pittosporum* leaves are thicker, with an average thickness of approximately 0.311 mm. Figure 3d shows the contents of chlorophyll carotenoid, water and dry matter. The average chlorophyll content of the 10 kinds of natural leaves is approximately $90.8 \, \mu {\text{g cm}}^{ - 2}$. The average chlorophyll content of *Magnolia* leaves is as high as ${135}{\text{.9 }}\mu {\text{g cm}}^{ - 2}$, while the average chlorophyll content in *Ginkgo* leaves is lower, only approximately $42.1 \, \mu {\text{g cm}}^{ - 2}$. Kinoshita et al. adopted *Ginkgo* leaves to study its photosynthesis characteristics, which also exhibit the characteristic of low chlorophyll content. They found that these *Ginkgo* leaves are less photosynthetic than other trees grown in Kyoto^69^. It is worth noting that in our previous work, the stomatal distribution characteristics of the abaxial surface of *Ginkgo* leaves were investigated, and it was found that compared with other plants in the same environment, the stomata of *Ginkgo* leaves were sparser and the size of a single stoma was smaller. The number of stomata per unit area is only approximately $59{\text{ mm}}^{ - 2}$, and the length of the long and short axes of the spindle-shaped stomata are approximately 12.49 and $4.91 \, \mu {\text{m}}$, respectively^70^. Therefore, the stomatal conductance of *Ginkgo* leaves is small, resulting in a low CO_2_ diffusion flux and net photosynthetic rate, and a low chlorophyll content required for photosynthesis. According to the analysis in Sect. “Convergent behavior of chlorophyll content”, it can be known that chlorophyll content significantly affects the visible light absorptance of the natural leaves. Figure 3d also shows the test results of carotenoid, water and dry matter contents in the natural leaves, respectively. The average contents are approximately $11.5 \, \mu {\text{g cm}}^{ - 2}$, $11.6{\text{ mg cm}}^{ - 2}$ and $9.9{\text{ mg cm}}^{ - 2}$, respectively, and the ratio of carotenoid content to chlorophyll content is approximately 0.13. Datt et al. and Wang et al. both show that the contents of these two kinds of pigments in natural leaves grown in the same environment has a certain proportional relationship^71,72^. It is worth noting that although the species of the 10 kinds of leaf samples are significantly different, the reflectance and transmittance of the leaves and the contents of each component show a convergent behavior. Ollinger pointed out that plants can optimize the relevant physiological parameters of resource acquisition and effective utilization in the evolutionary process, and the optimal combination of the physiological parameters in a certain environment is limited, thus the physiological characteristics of plants in the same environment tend to be consistent in the evolutionary process, and the combination of these physiological parameters reflects the intrinsic relationship between plant functional traits and the trade-off relationship for resource allocation^73^. According to this principle, Díaz and Meireles et al. utilized the changes of leaf spectral characteristics to investigate the evolution process of plant physiological characteristics^74,75^. From the analysis in the next paragraph, it can be seen that the leaves of different species grown in the same natural environment also exhibit similar structural parameters.

The structural parameters of the natural leaves can be inverted by using the above test results of the component contents and the solar spectral reflectance and transmittance of the natural leaves. Figure 3e shows the inversion results of structure parameters. It can be seen that the structure parameters of the 10 kinds of leaves are approximately in the range from 1.7 to 2.1. According to the study of larger scale of plant samples, most of the structural parameters of natural leaves are between 1.1 and 2.3^76^. According to the analysis in Sect. “Physiological significance of the similarity”, it can be seen that the structural parameters can also significantly affect the visible light absorptance of the natural leaves. Notably, structural parameters vary with leaf species and growth stages. The structural parameters of mature leaves of monocotyledonous plants are approximately in the range from 1.27 to 1.73, while dicotyledonous leaves have palisade layers and sponge layers, and the stacked mesophyll cells is more complex, thus the corresponding structural parameters are larger, and the structural parameters of mature leaves are approximately in the range from 1.71 to 2.21^77^. Demarez et al. found that the structural parameters of temperate deciduous plants gradually increase in the early stage of growth, remain basically constant in the mature stage, and increase rapidly in the senescence stage. The rapid increase of structural parameters during senescence stage is related to the enhanced scattering caused by the degradation of mesophyll structure due to the decrease of water content^78^. The above analysis shows that the structural parameter defined in the PROSPECT model can reflect the changing characteristics of the actual stacked mesophyll cells in the natural leaves.

### Formation mechanism of the similarity

In order to study the influence of the surface rough structure and interior stacked mesophyll cells on the similar characteristics of reflectance and transmittance, four cases were introduced in Sect. “Cases design with different leaf structures”. When calculating the reflectance and transmittance corresponding to the models, the contents of each component are set as the average values of the 10 kinds of leaf samples, and the specific values have been introduced in Sect. “Results of characterizations”. Figure 3f shows the calculation results of the reflectance and transmittance corresponding to Case-1. As can be seen, the reflectance and transmittance are significantly different. The reflectance is below 0.1 in the entire solar spectrum waveband, while the transmittance in the range from 800 to 1300 nm is as high as 0.9, and the similarity coefficient is approximately 0.34. Figure 3g shows the calculation results of the reflectance and transmittance corresponding to Case-2. The reflectance is significantly increased, showing similar characteristics to those of the natural leaves. This is because with the introduction of the surface rough structure, the collimated light entering the natural leaves from the adaxial surface is scattered into diffuse light within a certain angle range, and the corresponding interface reflectance is larger than that of parallel light. Interface reflection occurs when the light arrives at the abaxial interface, and more light is reflected back to the incident direction, thus the solar spectral reflectance is increased. However, the reflectance corresponding to Case-2 is approximately 0.35 in the range from 800 to 1300 nm, which is still significantly lower than the transmittance, and the similarity coefficient is 0.76. Figure 3h shows the calculation results of the reflectance and transmittance corresponding to Case-3, where the structure parameter is set as 1.8. It can be seen that after considering the scattering process in the stacked mesophyll cells of natural leaves, the reflectance and transmittance show significantly similar characteristics, which are consistent with our test results and the results in the literature^79^. The similarity coefficient is 0.97. Figure 3i shows the calculation results of the reflectance and transmittance corresponding to Case-4, which is corresponds to the leaf structures of the *Osmanthus* leaves. It can be seen that the reflectance and transmittance also show significantly similar characteristics, indicating that the scattering process of the stacked mesophyll cells in the leaf is the main reason for the significantly similar characteristics of reflectance and transmittance, instead of the the rough structures on the leaf surfaces. It is worth noting that compared with Case-4, the adaxial surface of leaf in Case-3 has a surface rough structure and higher interface reflectance, thus the reflectance in the near-infrared plateau region is slightly higher. In the following analysis, Case-1, Case-2 and Case-3 were mainly used to study the effects of stacked mesophyll cells, chlorophyll content and leaf thickness on the visible light absorptance.

### Natural optimization characteristics of natural leaves

#### Physiological significance of the similarity

**Figure 4:**
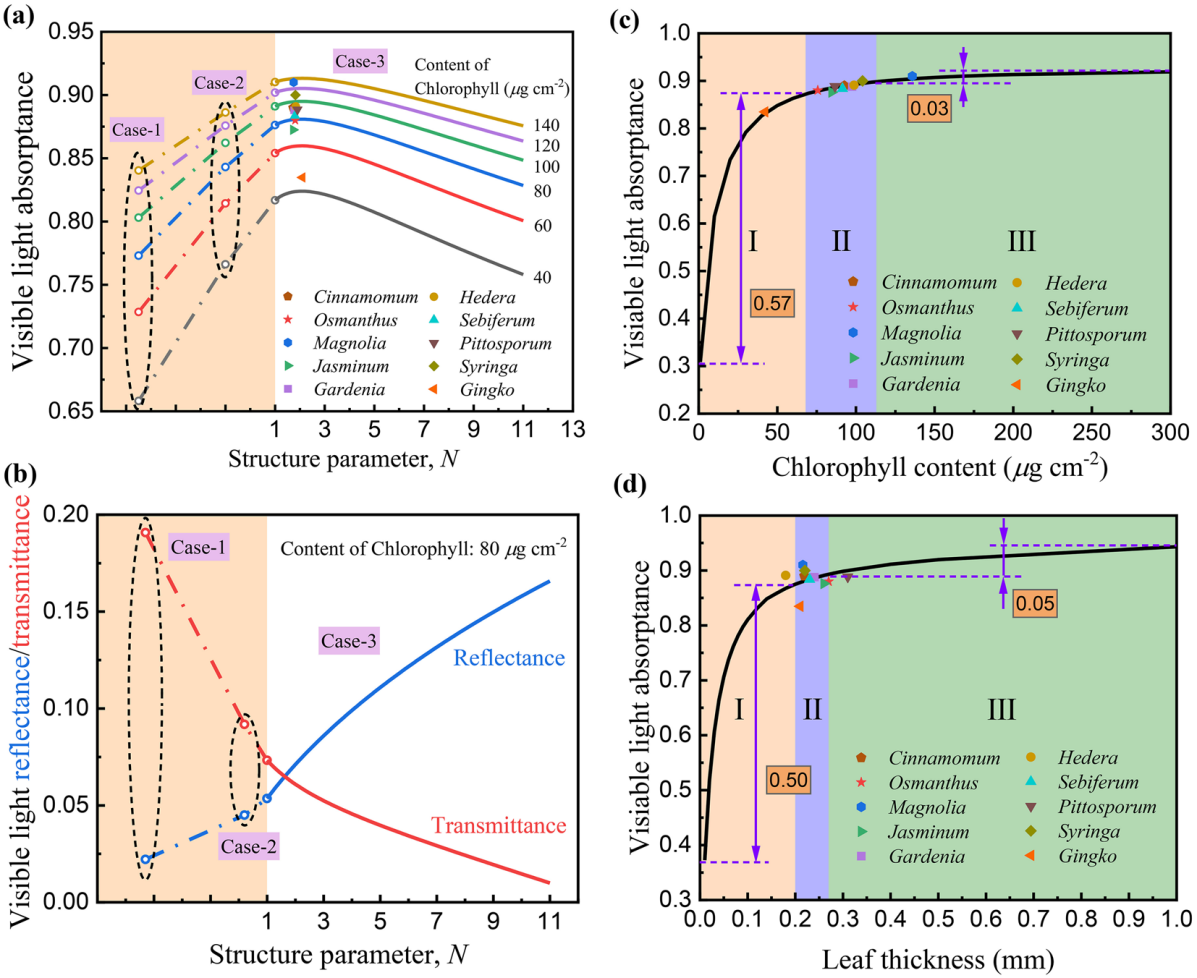
(**a**) Calculated visible light absorptance of leaves with different structures and chlorophyll contents. For the marked points of the natural leaves, the structure parameters were inversed with the radiation model, while the visible light absorptaces were measured. (**b**) Calculated visible light reflectance and transmittance of leaves with different structures. The chlorophll content was set as 80 μg cm^-2^. (**c**) Calculated visible light absorptance of natural leaves with different chlorophyll contents. (**d**) Calculated visible light absorptance of natural leaves with different leaf thicknesses.

It can be seen from Figs. 3f–h that the visible light transmittance corresponding to Case-1, Case-2 and Case-3 gradually decreases, and the corresponding visible light absorptance gradually increases, approximately 0.79, 0.85 and 0.88, respectively. In order to further analyze the influence of leaf structure characteristics on the visible light absorptance, the visible light absorptance of leaves with different structures were calculated with the radiation models. For Case-3, the structure parameter varies from 1 to 11, and a larger structure parameter represents a stronger scattering of incident radiation in the stacked mesophyll cells in the natural leaves. Figure 4a shows the visible light absorptance of the different cases. The hollow dots in the black dotted boxes in Fig. 4a are the calculated results of Case-1 and Case-2. They don't correspond to any structural parameters. They are shown in the same figure with Case-3 just to exbibit the change of visible light absorptance with different leaf structures. The visible light absorptance corresponding to Case-1 is low, while the visible light absorptance corresponding to Case-2 increases significantly. This is because that the incident radiation is scattered within a certain angle after entering the leaves, which increases the optical path inside the leaves and enhances the light absorption by chlorophyll. Thus, the visible light absorptance increases significantly, while the visible light transmittance is significantly reduced and the visible light reflectance gradually increases, as shown in Fig. 4b. After introducing the scattering process in the stacked mesophyll cells in Case-3, the interior scattering is further enhanced, resulting in a significant increase in the optical path, thus the visible light absorptance is significantly increased, and when the structure parameter is approximately 1.8, the visible light absorptance reaches a maximum. With the further increase of structure parameter, the scattering process continues to enhance, and more light is reflected back to the incident direction in this case, as shown in Fig. 4b, thus the visible light absorptance gradually decreases. It is worth noting that the abscissa of the intersection of reflectance and transmittance curves is approximately 1.8, indicating that under this structure parameter reflectance and transmittance are equal and exbibit similar characteristic. The above results show that there is a maximum characteristic between the visible light absorptance of natural leaves and the structural parameter. Moreover, it can be seen from Fig. 4a that the structural parameters of the 10 kinds of natural leaves are all distributed around 1.8, indicating that natural leaves exhibit the characteristic of tending to the maximum of visible light absorptance. Therefore, the formation mechanism of the similarity of reflectance and transmittance of natural leaves is the light scattering caused by the stacked mesophyll cells in the leaves, and the physiological significant is to prompt the visible light absorptance of natural leaves tend to the maximum, thereby promoting the absorption of PAR by the natural leaves in the natural environments.

#### Convergent behavior of chlorophyll content

It can be seen from Fig. 4a that the chlorophyll contents of the different kinds of leaves vary greatly, and the visible light absorptance increases significantly with the increase of chlorophyll content. In this section, the effect of chlorophyll content on the visible light absorptance of the natural leaves is further studied. Figure 4c shows the visible light absorptance of the natural leaves with different chlorophyll contents calculated by PROSPECT model, wherein the ratio of carotenoid content to chlorophyll content was set as 0.13, and the water and dry matter contents were both set as the average values of the 10 kinds of leaf samples. It is worth noting that the mean and standard deviation are commonly used mathematical models to describe the average and discrete characteristics of physiological parameters of plant communities, respectively^80,81^, therefore, in the following analysis, the above models were used to describe the distribution characteristics of chlorophyll content and leaf thickness of the 10 kinds of leaf samples. As can be seen in Fig. 4c, three regions can be divided according to the statistical distribution characteristics of the chlorophyll contents of the leaf samples, in which the center point of the region II is the average value of the chlorophyll contents, approximately $90.8 \, \mu {\text{g cm}}^{ - 2}$, and the width is twice the standard deviation of the chlorophyll contents, approximately $44.6 \, \mu {\text{g cm}}^{ - 2}$. It can be seen from the figure that with the increase of chlorophyll content, the visible light absorptance gradually increases, but the increasing rate gradually decreases, and most plants are distributed in region II. The left side of region II is region I, which corresponds to smaller chlorophyll content and lower visible light absorptance. *Ginkgo* with chlorophyll content of $42.1 \, \mu {\text{g cm}}^{ - 2}$ is in the region I, and the corresponding visible light absorptance is at a low level, which affects the photosynthesis and growth of *Ginkgo*^82^. It is worth noting that in Region I, the visible light absorptance increases significantly with the increase of chlorophyll content. When the chlorophyll content increased from 0 to $68.5 \, \mu {\text{g cm}}^{ - 2}$, the visible light absorptance increases from 0.31 to 0.88, and the magnitude of increase is 0.57. The right side of region II is region III. In this region, the visible light absorptance also increases gradually with the increase of chlorophyll content, but the increasing rate is significantly lower than that in region I. When the chlorophyll content increased from 113.1 to $300 \, \mu {\text{g cm}}^{ - 2}$, the visible light absorptance only increased from 0.89 to 0.92, and the magnitude of increase is only 0.03, indicating that when the chlorophyll content is too high, its influence on the visible light absorptance of natural leaves is significantly weakened. *Magnolia* is distributed in region III, with an average chlorophyll content of $135.9 \, \mu {\text{g cm}}^{ - 2}$, which is near the right boundary of region II. Noda et al. studied the relationship between the visible light absorptance and chlorophyll content of *Betula ermanii* and *Quercus crispula* through experiments, and found that when the chlorophyll content in the leaves gradually increased from 18 to $56 \, \mu {\text{g cm}}^{ - 2}$, the visible light absorptance increased from 0.72 to 0.89^40^, showing a similar change characteristic to that of the curve in Fig. 4c. It is worth noting that chlorophyll undergoes anabolism and catabolism in natural leaves, and nutrients and energy are consumed during the metabolic processes^83,84^, therefore, the chlorophyll content of natural leaves grown in a certain natural environment needs to be adjusted, resulting in achieving a higher visible light absorptance with a lower chlorophyll content. According to the leaf economic spectrum theory, the nutrients and energy consumed in the process of chlorophyll metabolism are "costs", and the PAR absorbed by natural leaves is "benefit". There is a trade-off relationship between chlorophyll content and visible light absorptance. More importantly, chlorophyll is a strong photosensitizer. When the chlorophyll content is too high, reactive oxygen species can be generated in the leaves and promote cell death^85^. Therefore, in order to achieve healthy growth and development, the metabolic processes of chlorophyll need to be controlled precisely. In summary, most natural leaves are distributed in region II, indicating that the chlorophyll content of natural leaves grown in a certain natural environment exhibits the characteristic of convergence in an optimal region, thereby optimizing visible light absorption process.

#### Convergent behavior of leaf thickness

Figure 4d shows the visible light absorptance of natural leaves with different leaf thicknesses calculated by PROSPECT model, wherein the contents of each component are set as the average values of the 10 kinds of leaf samples. Specifically, the average contents of chlorophyll, carotenoid, water and dry matter are approximately 4.22 mg/g, 0.53 mg/g, 0.54 g/g and 0.46 g/g, respectively. It can be seen from the figure that with the increase of thickness, the visible light absorptance gradually increases. It is worth noting that some markers of the leaf samples are far away from the curve in the ordinate direction, because the curve is calculated based on the average value of chlorophyll content, and the actual chlorophyll contents of the leaf samples vary greatly. Three regions can be divided according to the statistical distribution characteristics of leaf thickness. The center point of region II is the average thickness of approximately 0.24 mm, and the width is twice the standard deviation of the thickness, approximately 0.07 mm. It can be seen from the figure that the growth rate of visible light absorptance gradually decreases with the increase of leaf thickness, and most plants are distributed in region II. Region I is on the left side of region II, which corresponds to the smaller leaf thickness and lower visible light absorptance. The *Hedera* leaf is in region I, which is near the left border of region II, and the average leaf thickness is approximately 0.18 mm. It is worth noting that in Region I, the visible light absorptance increases significantly with increasing leaf thickness. When the leaf thickness increases from 0.01 to 0.20 mm, the visible light absorptance increases from 0.37 to 0.87, and the magnitude of increase is approximately 0.50. The right side of region II is region III. In this region, the visible light absorptance increases gradually with the increase of leaf thickness, but the growth rate is significantly lower than that in region I. When the leaf thickness increases from 0.27 to 1.00 mm, the visible light absorptance only increases from 0.89 to 0.94, and the magnitude of increase is only approximately 0.05, indicating that when the leaf thickness is too large, its influence on the visible light absorptance is significantly weakened. *Pittosporum* is distributed in region III, and the average thickness is approximately 0.31 mm, which is close to the right boundary of region II. The leaf is the organ of the plant for photosynthesis. With the increase of leaf thickness, the volume or number of mesophyll cells in the leaves increases^53,86^, causing an increase in nutrients needed to maintain physiological activities of the leaves^87^. In Region II, higher visible light absorptance can be achieved with smaller leaf thickness and less nutrient consumption. Most natural leaves are distributed in region II, indicating that the thickness of natural leaves growing in a certain natural environment exhibit a convergent trait of distributing in an optimal region, thereby optimizing visible light absorption process.

Terashima et al. constructed a leaf photosynthesis model based on the relationship between leaf energy balance and CO_2_ diffusion, and investigated the relationship between net photosynthesis rate and mesophyll thickness under different CO_2_ diffusion conductance^88^. It was found that for natural leaves with unilaterally distributed stomata, with the increase of mesophyll thickness, the net photosynthetic rate first increases rapidly and then decreases slowly, resulting in that the net photosynthetic rate shows a maximum characteristic. When the CO_2_ diffusion conductance from the cell wall surface to the chloroplast matrix is approximately $1 \times 10^{ - 3} {\text{ m/s}}$, the maximum of the net photosynthesis rate is approximately 18 μmol m^-2^ s^-1^, and the corresponding mesophyll thickness is approximately 0.25 mm. It is worth noting that the stomatal conductance corresponding to this CO_2_ diffusion conductance is approximately $1 \times 10^{ - 2} {\text{ m/s}}$, and the daytime stomatal conductance of the leaves in Wuxi summer is also approximately $1 \times 10^{ - 2} {\text{ m/s}}$, which was measured by the AP4 stomometer in our previous work of investigating the stomatal transpiration characteristics of the natural leaves^89^. Wuxi and Hefei are geographically close to each other and have similar climatic conditions. It can be approximately considered that the stomatal conductance of the natural leaves grown in these two places are close. Therefore, according to the photosynthesis model of natural leaves, the mesophyll thickness corresponding to the maximum net photosynthesis rate of the plants in Hefei summer is approximately 0.25 mm, which is basically consistent with the average leaf thickness of 0.24 mm obtained from the experimental test in this work. In addition to the theoretical analysis, experimental investigations were also conducted, and it was found that the maximum net photosynthesis rate is significantly related to leaf thickness^90,91^. Araus et al. studied the relationship between leaf thickness and net photosynthetic rate of different types of shade plants. During the experiments, the maximum PAR flux was set as 470 μmol m^-2^ s^-1^. It was found that the leaf thickness of *Fatsia* is approximately 192 μm, corresponding to the maximum net photosynthetic rate as high as 6.9 μmol m^-2^ s^-1^, while the leaf thickness of *Philodendron* is approximately 296 μm, and the corresponding maximum net photosynthetic rate is only approximately 2.6 μmol m^-2^ s^-1^^92^. The above results show that the net photosynthesis rate has a maximum characteristic with increasing of leaf thickness, and the convergent behavior of leaf thickness in natural environments makes the net photosynthesis rate approach the maximum.

## Conclusions

The formation mechanism and physiological significance of the similarity characteristic of the solar spectral reflectance and transmittance of natural leaves were investigated, and it was found that the light scattering caused by the stacked mesophyll cells is the reason for the similarity, and this scattering process makes the visible light absorptance of natural leaves approaches a maximum, thereby promoting PAR absorption in the natural environments. Interestingly, both the chlorophyll content and leaf thickness in the natural environment show a convergent behavior, which make the natural leaves exhibit the characteristics of high visible light absorptance, reflecting the PAR utilizing strategies of leaves grown in the natural environments. The analysis method of visible light absorption characteristics of natural leaves proposed in this work has theoretical prediction ability in photosynthesis improvement, and can be used to guide the research directions of crop optimization.

## Supplementary Information

Supplementary Information 1.

Supplementary Information 2.

## Data Availability

The datasets generated during and/or analyzed during the current study are available from the corresponding author on reasonable request. Please contact Y.H. if the data from this study is requested.
