# Supplementary material for: Light scattering in stacked mesophyll cells results in similarity characteristic of solar spectral reflectance and transmittance of natural leaves
Source: Sci Rep. 2023 Mar 22;13:4694. doi: 10.1038/s41598-023-31718-1 (PMC10033640; doi:10.1038/s41598-023-31718-1)
Supplement: Supplementary file 2 — Supplementary Information 2. [file 41598_2023_31718_MOESM2_ESM.docx]

**SUPPORTING INFORMATION FOR**

Light scattering in stacked mesophyll cells results in similarity characteristic of solar spectral reflectance and transmittance of natural leaves

Kai Xu, Hong Ye, *

Department of Thermal Science and Energy Engineering, University of Science and Technology of China, Hefei 230027, People’s Republic of China

*Correspondence to: H. Ye (E-mail: hye@ustc.edu.cn)

**Table of contents**

**1 Leaf Radiation Models**

1.1 Single-layer plate model

1.2 PROSPECT model

**References**

**1 Leaf Radiation Models**

**1.1** **Single-layer plate model**

The reflectance and transmittance of the single-layer plate can be calculated using ray tracing method:

$\rho=\rho_{12}+t_{12}\tau^{2}\rho_{21}t_{21}+t_{12}\tau^{4}\rho_{21}^{3}t_{21}+......\text{ =}\rho_{12}+\frac{t_{12}t_{21}\rho_{21}\tau^{2}}{1-\rho_{21}^{2}\tau^{_{2}}}$ (S1)

$t=t_{12}\tau t_{21}+t_{12}\tau^{3}\rho_{21}^{2}t_{21}+t_{12}\tau^{5}\rho_{21}^{4}t_{21}......\text{ =}\frac{t_{12}\tau t_{21}}{1-\rho_{21}^{2}\tau^{_{2}}}$ (S2)

where $t_{ij}$represents the interface transmittance when light is incident from medium *i* to medium *j*, and the corresponding interface reflectance is $\rho_{ij}=1-t_{ij}$, $\tau$ represents the transmittance when light passes through medium 2. When the light is incident from medium 1 to medium 2 at the incident angle $\theta_{1}$, the interface reflectance $\rho_{12}(\theta_{1})$ is [1]

$\rho_{12}=\frac{1}{2}\left\{\left[\frac{\sqrt{n^{2}-\sin^{2}\theta_{1}}-\cos\theta_{1}}{\sqrt{n^{2}-\sin^{2}\theta_{1}}+\cos\theta_{1}}\right]^{2}+\left[\frac{n^{2}\cos\theta_{1}-\sqrt{n^{2}-\sin^{2}\theta_{1}}}{n^{2}\cos\theta_{1}+\sqrt{n^{2}-\sin^{2}\theta_{1}}}\right]^{2}\right\}$ (S3)

where *n* is the refractive index of the plate. The rough surface of natural leaves has many oblique surfaces, as shown in Figure 2 in the manuscript. When the light is incident vertically, the incidence angle of light for one of the inclined surfaces is $\alpha$. The relationship between the probability density function *D*, the surface roughness parameter $\sigma$ and incidence angle $\alpha$ is as follows [2]:

$D(\alpha,\sigma)=\frac{1}{\cos^{4}\alpha}\exp(-(\frac{\tan\alpha}{\sigma})^{2})$ (S4)

The surface roughness parameter $\sigma$ of most plants is less than 0.5. As can be seen from Figure S1, when $\sigma$ is in the range from 0 to 0.5, the incident angle $\alpha$ is approximately in the range from 0° to 40°. Thus, the maximum of incident angle $\alpha$ is set as 40° to calculate the upper surface interface reflectance of the natural leaves.

**Figure S1.** Surface element inclination probability density distribution function.

When the light is incident perpendicular to the surface of the plate, the maximum incident angle is defined as $\alpha$, then the upper surface interface reflectance is

(S5)

where is the azimuth angle. After passing through the plate at the refraction angle $\theta_{2}$ and reaching the lower surface, the light transfers from medium 2 to medium 1. If $\theta_{2}$ is greater than the critical angle$\theta_{\text{c, 2}}[\arcsin(1/n)]$, total reflection occurs at the interface, that is $\rho_{21}\left(\theta_{2}\right)=1$, thus the lower surface interface reflectance is

(S6)

where $\rho_{21}(\theta_{2})$ is equal to $\rho_{12}(\theta_{2})$. It is worth noting that the travel distance of the light passing through the medium 2 with thickness *D* in a single time is $D/\cos\theta_{2}$, thus the transmittance of the single-layer plate is

(S7)

where $K_{\text{p}}(\text{mm}^{-1})$ is the absorption coefficient per unit thickness, which can be expressed as

$K_{\text{p}}\text{=}\frac{\sum K_{i}C_{i}}{D}$ (S8)

where $K_{i}$ is the absorption coefficient of component *i* in leaves, and $C_{i}$ is the content of component *i* per unit area of leaves.

**1.2 PROSPECT model**

From the perspective of principle, the multi-layer structure obtained by PROSPECT model can be used to reflect the scattering characteristics of the stacked cell structure in natural leaves equivalently. From the perspective of calculation, it is convenient to calculate the reflectance and transmittance of the multi-layer structure, because all but the bottom layer have the same thickness. In addition, the division method of layers specified in PROSPECT model is generally accepted. Only *N* obtained by this method can be called the structural parameter which belongs to the terms of plant spectroscopy. Therefore, the division method of layers specified in PROSPECT model was utilized in this work although there can be other methods. The collimated light entering the leaf transfers to diffused light, and the range of $\theta_{2}$ is adjusted from $0^{\circ}$ to $90^{\circ}$ to manifest the scattering effect in the natural leaves. Reflectance and transmittance of the entire *N* layers are

$R_{N}\text{ =}\rho_{1}+\text{ }t_{1}R_{N-1}t_{N-1}+t_{1}\rho_{N-1}R_{N-1}^{2}t_{N-1}+......\text{=}\rho_{1}+\frac{t_{1}R_{N-1}t_{N-1}}{1-\rho_{N-1}R_{N-1}}\text{ }$ (S9)

and

$T_{N}\text{ =}t_{1}T_{N-1}+\text{ }t_{1}R_{N-1}\rho_{N-1}T_{N-1}+t_{1}R_{N-1}^{2}\rho_{N-1}^{2}T_{N-1}+......\text{=}\frac{t_{1}T_{N-1}}{1-\rho_{N-1}R_{N-1}}\text{ }$ (S10)

respectively, where $\rho_{1}$ and $t_{1}$ are the reflectance and transmittance of the first layer, $\rho_{N-1}$ and $t_{N-1}$ are the reflectance and transmittance of each remaining layers, and $R_{N-1}$ and $T_{N-1}$are the total reflectance and transmittance of the $N-1$layers respectively. Stokes proposed the relationship between reflectance and transmittance of *N* layers homogeneous mediums [3]:

$\frac{R\left(N\right)}{b^{N}-b^{-N}}\text{ =}\frac{T\left(N\right)}{a-a^{-1}}=\frac{1}{ab^{N}-a^{-1}b^{-N}}\text{ }$ (S11)

where $R(N)$and $T(N)$are the reflectance and transmittance of the *N* layers homogeneous mediums, respectively. For the PROSPECT model, the remaining $N-1$layers except the first layer are homogenous mediums, thus according to Equation S11,

$R_{N-1}\text{ }=\frac{b^{N-1}-b^{1-N}}{ab^{N-1}-a^{-1}b^{1-N}}\text{ }$ (S12)

$T_{N-1}\text{ }=\frac{a-a^{-1}}{ab^{N-1}-a^{-1}b^{1-N}}$ (S13)

The reflectance and transmittance of the *N* layers can be obtained by substituting Equation S12 and S13 into Equation S9 and S10:

$R_{N}\text{ = }\rho_{1}+(t_{1}\frac{b^{N-1}-b^{1-N}}{ab^{N-1}-a^{-1}b^{1-N}}t_{N-1})/(1-\rho_{N-1}\frac{b^{N-1}-b^{1-N}}{ab^{N-1}-a^{-1}b^{1-N}})$ (S14)

$T_{N}\text{ = }\frac{t_{1}\left(a-a^{-1}\right)}{ab^{N-1}-a^{-1}b^{1-N}-\rho_{N-1}\left(b^{N-1}-b^{1-N}\right)}\text{ }$ (S15)

where

$a=\left(1+\rho_{N-1}^{2}-t_{N-1}^{2}+\delta\right)/2\rho_{N-1}$ (S16)

$b=\left(1-\rho_{N-1}^{2}+t_{N-1}^{2}+\delta\right)/2t_{N-1}$ (S17)

$\delta=\sqrt{\left(t_{N-1}^{2}-\rho_{N-1}^{2}-1\right)^{2}-4\rho_{N-1}^{2}}$ (S18)

**References:**

[1] R. Molenaar, J. J. ten Bosch, J. R. Zijp, Determination of Kubelka-Munk scattering and absorption coefficients by diffuse illumination, Applied Optics 38 (1999) 2068-2077.

[2] J.-B. Feret, C. François, G. P. Asner, A. A. Gitelson, R. E. Martin, L. P. Bidel, S. L. Ustin, G. le Maire, S. Jacquemoud, PROSPECT-4 and 5: Advances in the leaf optical properties model separating photosynthetic pigments, Remote Sensing of Environment 112 (2008) 3030-3043.

[3] L. B. Tuckerman, On the intensity of the light reflected from or transmitted through a pile of plates, Journal of the Optical Society of America 37 (1947) 818-825.
